# Elevational Variation in Rhizosphere Bacterial Assembly and Fine-Scale Taxon Differentiation of *Carex enervis* in Arid and Semi-Arid Alpine Meadows

**DOI:** 10.3390/microorganisms14071468

**Published:** 2026-07-03

**Authors:** Baokang Yang, Junfang Zhou, Xuemin He

**Affiliations:** 1 College of Ecology and Environment, Xinjiang University, Urumqi 830017, China; 107552405052@stu.xju.edu.cn (B.Y.); zhoujunfangnzs@163.com (J.Z.); 2 Key Laboratory of Oasis Ecology of Education Ministry, Xinjiang University, Urumqi 830017, China; 3 Xinjiang Jinghe Observation and Research Station of Temperate Desert Ecosystem, Ministry of Education, Urumqi 830017, China; 4 Technology Innovation Center for Ecological Monitoring and Restoration of Desert-Oasis, Ministry of Natural Resources, Urumqi 830001, China

**Keywords:** *Carex enervis*, rhizosphere microorganisms, assembly mechanisms, micro-niche differentiation

## Abstract

Unraveling rhizosphere microbial assembly and plant–microbe co-adaptation is essential for understanding how fragile mountain ecosystems respond to environmental stress. This study investigated the rhizosphere bacterial communities of *Carex enervis* C. A. Mey, a dominant species in arid and semi-arid alpine meadows, along an altitudinal gradient from 1160 to 1860 m. By integrating high-throughput sequencing, iCAMP-based community assembly analysis, niche differentiation assessment, and partial least squares path modeling, we examined associations among macro-environmental gradients, rhizosphere soil conditions, bacterial community assembly, and ammonium nitrogen availability. The results revealed a dual-track assembly pattern. Macro-environmental heterogeneity, particularly in elevation and precipitation, was associated with rare microbial diversity primarily through heterogeneous selection. In contrast, abundance-weighted patterns suggested homogeneous selection of core dominant microbial groups in the rhizosphere. Within several dominant genera, closely related taxa showed divergent covariation patterns rather than uniform responses along the environmental gradient, suggesting potential fine-scale differentiation in environmental responses. Path analysis further indicated that enzyme-based rhizosphere activity proxies were associated with the relative abundance of microbial response groups and with the availability of ammonium nitrogen. These findings suggest that the rhizosphere conditions of *Carex enervis* are associated with bacterial assembly patterns, fine-scale taxon differentiation, and nutrient-related soil variables along the elevational gradient. This study provides new insight into plant–microbe co-adaptation in arid and semi-arid mountain ecosystems.

## 1. Introduction

Due to their environmental gradients and highly heterogeneous habitat conditions, mountain ecosystems are regarded as natural laboratories for testing fundamental ecological theories and elucidating the mechanisms underlying biodiversity formation [1,2,3]. Altitudinal gradients integrate systematic changes in multiple environmental factors—including temperature, precipitation, radiation, soil moisture, and nutrient availability—and exert a profound influence on the structure, function, and stability of biological communities [4,5]. Compared to macroorganisms, soil microorganisms—due to their short life cycles, rapid metabolic responses, and high sensitivity to environmental changes—are considered among the biological groups that most directly reflect the effects of ecological gradients [6,7].

Understanding the rules governing community assembly has long been a central issue in microbial ecology [8,9,10]. Community ecology theory posits that the formation of microbial community structure is driven by both deterministic processes (such as environmental filtering and interspecific interactions) and stochastic processes (such as diffusion constraints and ecological drift) [11,12,13]. Classical niche theory emphasizes the central role of environmental selection in shaping community structure. In contrast, neutral theory suggests that stochastic processes can also dominate community assembly under conditions of minimal resource differentiation or diffusion constraints [14,15,16]. In recent years, with the advancement of high-throughput sequencing and sophisticated null models (such as the iCAMP framework), researchers have come to recognize that deterministic factors (such as heterogeneous and homogeneous selection) and stochastic factors are not mutually exclusive, but rather exhibit continuous variation—and even nonlinear transitions—along environmental gradients [17,18,19,20].

As the microenvironment where soil–plant–microbe interactions are most active, the plant rhizosphere plays a key role in regulating the structure and function of microbial communities [21]. Root exudates provide a stable and selective carbon source for microorganisms, causing the rhizosphere microbial community to deviate significantly from the background of the surrounding non-rhizosphere soil, thereby becoming a “hotspot” where environmental filtration and biological interactions are highly coupled [22]. In mountain meadow ecosystems, dominant plants may be associated with relatively stable rhizosphere microhabitats and the enrichment of core microbial taxa, thereby potentially buffering part of the spatial heterogeneity of the external environment [23]. However, under limited rhizosphere resources, closely related species with highly overlapping basic niches inevitably face intense competition [24,25,26]. How these species achieve coexistence along environmental gradients through microhabitat differentiation strategies and how such shifts in micro-ecological community assembly cascade to influence plant uptake of readily available nutrients (such as available nitrogen) remain poorly understood due to a lack of systematic quantitative assessments and comparative studies.

The mountainous meadows in southern Hutubi County, Xinjiang, are located in an arid and semi-arid region and are highly sensitive to climate change and environmental disturbances. *Carex enervis* C. A. Mey, the dominant species in this region at elevations ranging from 1160 m to 1860 m, provides an ideal model for studying the response of rhizosphere microorganisms to elevation gradients, owing to its stable population structure and adaptive characteristics. The selection of *Carex enervis* also allowed us to minimize variation due to host identity, because this species occurs continuously across the sampled elevational gradient and is a common dominant in rhizosphere habitats in the local alpine meadow ecosystem. Based on this, this study focuses on the rhizosphere soil of *Carex enervis* distributed along an altitudinal gradient (1160 m to 1860 m). Combining high-throughput sequencing, the iCAMP assembly framework, intragenus covariation analysis, and partial least squares path modeling (PLS-PM), this study aimed to address the following questions: (1) How are environmental gradients and rhizosphere conditions associated with bacterial community assembly and fine-scale differentiation among closely related taxa? (2) How are rhizosphere activity proxies and microbial response groups associated with cascade responses involved in nutrient uptake, particularly ammonium nitrogen availability?

## 2. Materials and Methods

### 2.1. Study Area and Soil Sampling

**Figure 1:**
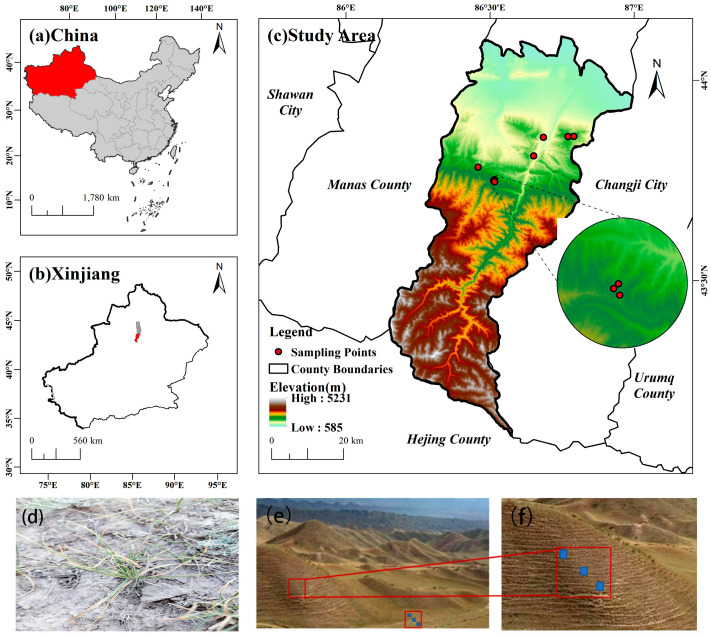
Overview of the study area and distribution of sampling sites: (**a**–**c**) location of the study area; (**d**) *Carex enervis* individual used for rhizosphere soil sampling; (**e**,**f**) locations of sampling plots.

The study area is located in the southern mountainous meadows of Hutubi County, Xinjiang (86°5′–87°8′ E, 43°7′–45°20′ N). The region has a temperate, continental, arid-to-semi-arid climate, with an average annual temperature of 2.9–7.1 °C and annual precipitation of 110–400 mm. Mountain meadows, with common species including *Carex enervis* C. A. Mey, *Salsola rosacea* L., and *Halimocnemis villosa* Kar, dominate the vegetation (Table S1). Plots were established across the study area along an elevation gradient (1160 m to 1860 m, at 100 m intervals) (Figure 1). This elevational gradient design was used to capture continuous natural variation in climate and soil conditions across the alpine meadow landscape. In contrast, using the same dominant host species across elevations reduced host-species-related variation in rhizosphere bacterial communities. Field sampling was conducted during the local growing season from July to August 2023, when *Carex enervis* plants were actively growing. To account for potential effects of microtopography on rhizosphere bacterial communities, three 1 m × 1 m herbaceous plots were randomly established on flat terrain, and three additional 1 m × 1 m plots were established on sloped terrain at each elevation (Figure 1e,f). Thus, six plots were sampled at each elevation, for a total of 48 plots across the eight elevations. The rhizosphere soil was defined as the soil tightly adhering to the root surface within approximately 1 mm of it. Within each plot, three similarly growing *Carex enervis* individuals were selected (Figure 1d), and their roots were excavated together with the attached soil. The rhizosphere soil from the three individuals in the same plot was gently brushed off with a sterile soft-bristled brush and pooled into a single composite rhizosphere soil sample. Therefore, each composite sample represented one biological replicate. In total, 48 composite rhizosphere soil samples were obtained. Each composite sample was divided into two subsamples: one subsample was stored at −80 °C for bacterial community analysis, and the other was air-dried indoors for soil physicochemical and enzyme activity measurements. Temperature and precipitation data for each sampling site were obtained from the WorldClim database.

### 2.2. Analysis of Soil Physicochemical Properties

Soil water content (SWC) was determined using the oven-drying method [27]. Soil pH was determined using the potentiometric method with a calibrated pH meter [28]. Electrical conductivity (EC) was measured using a conductivity meter [29]. Soil organic carbon (SOC) was determined using the high-temperature external heating potassium dichromate oxidation-volumetric method [30]. Nitrate nitrogen (NO_3_^−^-N) was measured using the phenol disulfonic acid colorimetric method. Ammonium nitrogen (NH_4_^+^-N) was measured using the extraction–indophenol blue colorimetric method [31]. Total nitrogen (TN) in soil was determined using the Kjeldahl distillation titration method. Total phosphorus (TP) in soil was determined using the sodium hydroxide fusion–molybdenum–antimony complexometric method. Available phosphorus (AP) in soil was determined using the hydrochloric acid–ammonium fluoride extraction–molybdenum–antimony complexometric method [32].

Methods for determining soil enzyme activity: In this study, the activities of soil alkaline phosphatase (AKP), soil urease (UE), and soil sucrase (SC) were all measured using enzyme-linked immunosorbent assay (ELISA) kits from Suzhou Mengxi Biomedical Technology Co., Ltd. (Suzhou, China). All soil samples were air-dried naturally or in a 37 °C oven and then sieved through a 50-mesh sieve. Soil alkaline phosphatase is based on the reaction principle of enzymatic hydrolysis of disodium phenylphosphate to release phenol under alkaline conditions. Using 0.1 g of air-dried soil as the test material, the enzymatic reaction is terminated after incubation at 37 °C for 24 h, and the reaction products are quantified colorimetrically at 660 nm. Enzyme activity units are defined as 1 unit per gram of soil per day at 37 °C, corresponding to the release of 1 μmol of phenol. Soil urease activity was determined using the indophenol blue colorimetric method, based on the principle of the enzymatic hydrolysis of urea to release ammonium nitrogen. A 0.05 g air-dried soil sample was used as the test material, with a control tube set up simultaneously. After incubation at 37 °C for 24 h, the supernatant was diluted 10-fold and quantified colorimetrically at 578 nm. Enzyme activity units are defined as 1 unit of enzyme activity per gram of soil sample per day at 37 °C, corresponding to the production of 1 μg of NH_3_^-^N. The soil sucrase assay is based on the principle of enzymatic hydrolysis of sucrose to produce reducing sugars, which then undergo a colorimetric reaction with 3,5-dinitrosalicylic acid. A 0.05 g air-dried soil sample is used as the test material, with a control tube set up simultaneously. After incubation at 37 °C for 24 h, the samples are developed in a 95 °C water bath for 5 min, and colorimetric quantification is performed at 510 nm. One enzyme activity unit is defined as the production of 1 mg of reducing sugars per gram of soil sample per day at 37 °C. All enzyme activity measurements are strictly quantified using the standard curve and calculation formula provided with the assay kit.

### 2.3. DNA Extraction, Amplification, and Illumina Sequencing Analysis

Total DNA was extracted from 0.5 g of fresh soil samples using the FastDNARSPIN Kit (MP Biomedicals, Santa Ana, CA, USA) according to the kit manual. Using a PCR thermal cycler (ABI GenAmp R9700, Foster City, CA, USA), the V3–V4 regions of the bacterial 16S rRNA gene were amplified using primers 341F (5′-CCTACGGGNGGCWGCAG-3′) and 806R (5′-GGACTACHVGGGTWTCTAAT-3′). PCR amplification was performed using an A200 thermal cycler (LongGene, Hangzhou, China). Each 50 μL PCR reaction contained 25 μL of 2× ES Taq MasterMix (Dye) (CoWin Biosciences, Taizhou, China), 2 μL of forward primer (10 μM), 2 μL of reverse primer (10 μM), 10 ng of template DNA, and ddH_2_O to a final volume of 50 μL. The PCR program was as follows: initial denaturation at 94 °C for 2 min, followed by 30 cycles of denaturation at 94 °C for 30 s, annealing at 55 °C for 30 s, and extension at 72 °C for 30 s, with a final extension at 72 °C for 10 min and holding at 4 °C. Three technical PCR replicates were performed for each sample. PCR products were checked by 1% agarose gel electrophoresis, purified, quantified, pooled in equimolar amounts, and sequenced on the Illumina MiSeq platform (Illumina, San Diego, CA, USA).

### 2.4. Sequencing Data Processing

Comprehensive quality control and analysis of the raw sequencing data were performed using the QIIME 2 platform. To ensure the accuracy and high resolution of downstream analysis results, the DADA2 algorithm was used to extract amplicon sequence variants (ASVs). First, the pooled library was disassembled using barcodes to obtain raw sequences for individual samples. Subsequently, the ‘dada2 denoise-paired’ plugin in QIIME 2 was invoked to filter and denoise the data. The denoised ASV representative sequences were subsequently clustered into operational taxonomic units (OTUs) at 99% sequence identity using VSEARCH v2.28.1. This ASV-to-OTU clustering step was performed to obtain stable operational units for downstream ecological analyses, particularly iCAMP-based phylogenetic binning and intragenus comparisons among closely related taxa. Because short 16S rRNA V3–V4 amplicons have limited resolution for reliable species- or strain-level assignment, the 99%–identity OTUs were treated as operational ecological units rather than as species-level taxa. Therefore, taxonomic interpretation in this study was mainly restricted to the genus level or to intragenus OTU-level response patterns. For species taxonomic annotation, this study utilized the latest Silva database (Release 138) as a reference. To improve annotation accuracy, primer pairs targeting the V3-V4 hypervariable regions of the bacterial 16S rRNA gene were first extracted and used to train the reference database. Subsequently, the classify-sklearn machine learning algorithm within the feature-classifier module of QIIME 2 was employed to align and annotate the obtained OTU representative sequences with species-level taxonomic information.

To provide a cautious functional context for the bacterial community data, predicted functional profiles were inferred from the 16S rRNA gene dataset using PICRUSt2 v2.5.1. Representative sequences and the corresponding OTU abundance table were used as input files. PICRUSt2 was used to place sequences into a reference phylogeny, normalize feature abundances by predicted 16S rRNA gene copy number, and infer KEGG Orthology (KO) abundances. Nitrogen metabolism-related KOs were then extracted according to the KEGG nitrogen metabolism pathway, including KOs associated with ammonia assimilation, nitrate reduction, nitrite reduction, denitrification, nitrogen transport, and nitrogen fixation. The predicted abundances of nifH, nifD, and nifK were included only as indicators of putative nitrogen fixation-related functional potential. These PICRUSt2 results represent predicted genomic potential inferred from 16S rRNA gene composition and do not indicate gene expression, enzyme activity, or directly measured nitrogen fixation.

### 2.5. Analysis of Niche Preferences and Environmental Factors

To test whether taxa within a given genus can share an altitudinal niche, we used the ‘propr’ v5.1 package. The raw data were converted into ratios, and for all ratios between Taxon A and Taxon B, we measured the proportionality of variation, Rho. The results were then filtered, with a final FDR estimate of 5%. Within each genus, we compared Rho values (a proxy for ecological niche similarity) between each OTU and nucleotide divergence to identify trends in niche correlation. We used linear models to test which genera showed a significant correlation between nucleotide divergence and Rho (*p* ≤ 0.05). We analyzed all genera (32 in total). For most of these groups, using the V3 and V4 hypervariable regions of the 16S rRNA gene, a nucleotide difference of 6 corresponds to a median sequence identity of 98.5% between two pairs. This nucleotide distance served as our threshold for considering two OTUs to be closely related. Finally, we tested which measured environmental parameters drove the patterns observed among closely related taxa. We used the vaclus function in the Hmisc package v5.2 in R v4.5.2 to assess the redundancy of environmental variables. We removed highly correlated variables (Spearman’s ρ^2^ > 0.7) from the MRM (e.g., TN, TOC, SWC, MAT, MAP). To model each OTU across different parameters, we used the corncob v0.4 package.

### 2.6. The Relationship Between the Assembly Process and Environmental Factors

Using a phylogeny-based framework (iCAMP) to analyze microbial assembly mechanisms has demonstrated superior quantitative performance. Since different taxonomic groups within the same community may result from distinct assembly processes, iCAMP classifies observed taxa into phylogenetic groups (BINs) and identifies the assembly processes governing the turnover of each bin, similar to QPEN. iCAMP estimates the relative importance of each assembly process, which is converted into a log-transformed ratio to avoid issues with compositional data. Subsequently, the Mantel test and cross-validation are used to analyze the correlation between the transformed values of each process and each log-transformed environmental variable [33]. A linear model with cross-validation is employed to assess pairwise correlations among environmental variables. Cross-validation employed a Monte Carlo approach, with 90% of the data used for training and 10% for testing. Significant associations (R^2^_CV_ > 0.01 and *p* < 0.05) were used to visualize correlations. Multicollinearity was accounted for. The “OPLS” function in the ropls package v1.42 was used to assess the contribution of environmental variables to each process. In the OPLS output, R^2^_Y_ and R^2^_X_ represent the percentages of variance in Y (the relative importance of each process) and in X (environmental variables) explained by the model. Q^2^_Y_ reflects the model’s overall predictive performance, as calculated via cross-validation. If P_Q_^2^_Y_ > 0.05, the model is considered overfitted.

### 2.7. Associations Among Rhizosphere Activity Proxies, Microbial Response Groups, and Nutrient Availability

To investigate microbial response patterns associated with rhizosphere conditions of *Carex enervis* C. A. Mey, this study screened for taxa associated with enzyme-based rhizosphere activity proxies using differential abundance tests. Using the R package corncob v0.4.0 to construct generalized linear models based on the beta-binomial distribution, we independently tested the direction and extent of responses of each operational taxonomic unit (OTU) to key proxy indicators of rhizosphere activity (such as soil urease UE, alkaline phosphatase AKP, and sucrase SC), while controlling for excessive dispersion caused by relative abundance. Based on coefficient estimates from differential analysis, we identified patterns of niche differentiation within core genera (e.g., *Kaistobacter* and *Rubrobacter*). OTUs within the same genus that exhibited significant positive associations (FDR < 0.05) with enzyme-based proxies of rhizosphere activity were aggregated and defined as “positively associated taxa,” representing microbial groups enriched under conditions of higher rhizosphere activity. OTU clusters exhibiting negative or non-significant associations were defined as “negatively associated or less responsive taxa,” representing microbial groups with lower relative abundance or weaker responses under conditions of higher rhizosphere activity.

To quantify the relative abundance of ecological strategy groups, we summed the relative abundances of the two functional response groups for each sample. Additionally, to eliminate potential biases in the estimation of path coefficients in subsequent models caused by the vast differences in units of measurement among macroclimate (altitude), soil physicochemical properties, soil enzyme activity, and microbial relative abundance, all data matrices included in the final analysis were pre-standardized using Z-scores (mean = 0, standard deviation = 1). Partial Least Squares Path Modeling (PLS-PM) was employed to evaluate hypothesized pathways of association among elevation, enzyme-based proxies of rhizosphere activity, microbial response groups, and ammonium nitrogen availability. The prior logical framework of the directed acyclic graph comprises five core components (latent variables/observed variables): (i) elevation, representing macro-environmental drivers; (ii) plant activity, a reflective latent variable composed of SC, UE, and AKP, used to characterize the intensity of rhizosphere interactions; (iii) assembly process (HeS), which exhibits the most pronounced response to environmental fluctuations; (iv) positively associated taxa; (v) negatively associated taxa; and (vi) available nitrogen (N, represented by NH_4_), indicating the final nutrient cycling outcome of the interaction process.

Model fitting was performed using the R package plspm v0.6. The internal consistency of the latent variables was verified using outer loadings (outer loadings > 0.7). Significance tests for path coefficients and the coefficient of determination (R^2^) were calculated using 1000 bootstrap resamples. The model’s overall predictive ability was assessed using the overall goodness of fit (GoF); a GoF value greater than 0.36 was considered indicative of a good model fit.

### 2.8. Statistical Analysis

All analyses were performed using R v4.5.2. Data processing was conducted using the phyloseq v1.54 and tidyverse v2.0 packages, and visualization was performed using ggplot2 v4.0. We defined abundant taxa as those with a relative abundance of 1% or higher in at least one sample. OTUs consistently falling below this threshold were considered permanently rare. For these two abundance groups, three OTU categories were defined based on occurrence frequency: widespread (occurrence rate ≥ 75%), intermediate (in >10% and <75% of samples), and narrow (in ≤10% of samples). Abundant OTUs were further identified as conditionally rare taxa (CRTs), i.e., taxa typically of low abundance but occasionally prevalent (bimodality = 0.9, relative abundance ≥ 1%). Since the assumptions of parametric tests (e.g., normality, equal variance, etc.) were not met in many analyses, we employed bootstrapping, permutation tests, and Mantel tests for comparative and association analyses. We conducted cross-validation to enhance the reliability of the results.

## 3. Results

### 3.1. Taxonomic Composition Characteristics of OTUs with Different Distribution Patterns

**Figure 2:**
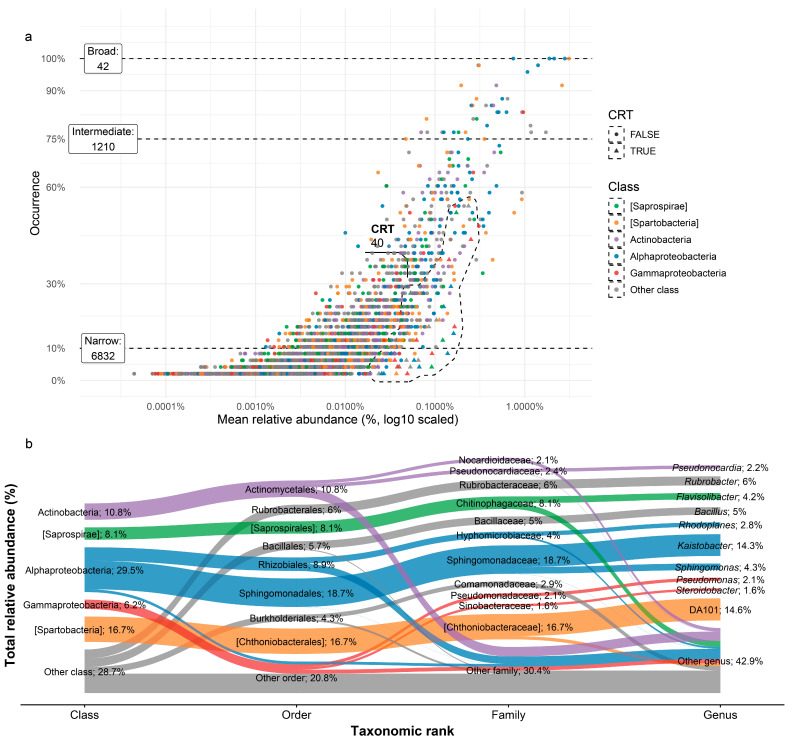
Occurrence–abundance distribution and overall taxonomic composition of rhizosphere bacterial OTUs. (**a**) Distribution of OTUs according to occurrence frequency and mean relative abundance across all samples. Dashed lines indicate broad, intermediate, and narrow distribution categories, and conditionally rare taxa (CRTs) are highlighted. (**b**) Alluvial plot showing the total relative abundance of bacterial taxa pooled across all samples and elevations at the class, order, family, and genus levels. Panel (**b**) provides an overall taxonomic overview of the OTUs included in panel (**a**) and is not intended to represent elevation-specific variation.

A total of 8124 OTUs (99% consensus) were detected across the 8 elevation gradient datasets. The distribution of OTUs was compared based on occurrence rates (narrow: occurrence rate ≤ 10%; intermediate: >10% and <75%; widespread: ≥75%) and abundance (abundant and rare, i.e., <1% across all samples). Most OTUs (84%) exhibited a narrow distribution, while only 42 OTUs showed a widespread distribution, 13 of which belonged to the rare category (Figure 2a).

Taxonomically, 16 of the broad OTUs belong to the Alphaproteobacteria, and 10 OTUs belong to the Spartobacteria. The 1210 moderate OTUs belong to 12 different phyla, with the dominant phyla being Proteobacteria (379 OTUs), Verrucomicrobia (240 OTUs), Actinobacteria (236 OTUs), and Bacteroidetes (209 OTUs). We also assessed whether rare OTUs occasionally became abundant (conditionally rare taxa, CRT) and identified 40 such OTUs. Alphaproteobacteria (8 OTUs) and Gammaproteobacteria (9 OTUs) were the most common CRTs (Figure 2b). Because Figure 2b summarizes taxa pooled across all samples and elevations, it was used only to provide an overall taxonomic background for the occurrence–abundance classification, rather than to evaluate elevation-specific differences in community composition.

Regarding alpha diversity, estimates of alpha richness were observed to follow a pattern of first decreasing and then increasing across the entire elevation gradient, with the lowest estimate (mean: 465.8) occurring at 1360 m. Regarding community similarity (beta diversity), the three major elevation clusters—low, middle, and high elevation—were completely distinct. Community structure showed succession with increasing elevation, indicating that elevation is the primary driver of bacterial community differentiation in this region (Figure S1). SIMPER analysis identified the key taxonomic units driving community dissimilarity: differences in the bacterial community were primarily attributed to changes in the abundance of OTU3980 (genus DA101, 34.91%), OTU193 (genus *Bacillus*, 32.35%), and OTU318 (genus DA101, 29.74%) (Table S2).

### 3.2. Variability in Niche Preferences Within a Microbial Genus

Here, the niche of a given taxon is defined as a set of environmental conditions that fluctuate along this altitudinal gradient and that allow for the growth or persistence of microorganisms. Co-occurrence and covariation suggest a possible niche similarity or mutualistic relationship. In the analysis, focusing on intragenus variability, the Rho metric (the proportion of variation shared between two taxa) serves as an alternative indicator for testing niche overlap among closely related taxa; it can be expressed as a function of nucleotide divergence between two sequences. As nucleotide distance increases, a decrease in Rho implies that the two taxa exhibit reduced covariance, behaving less similarly as they become more phylogenetically distinct.

**Figure 3:**
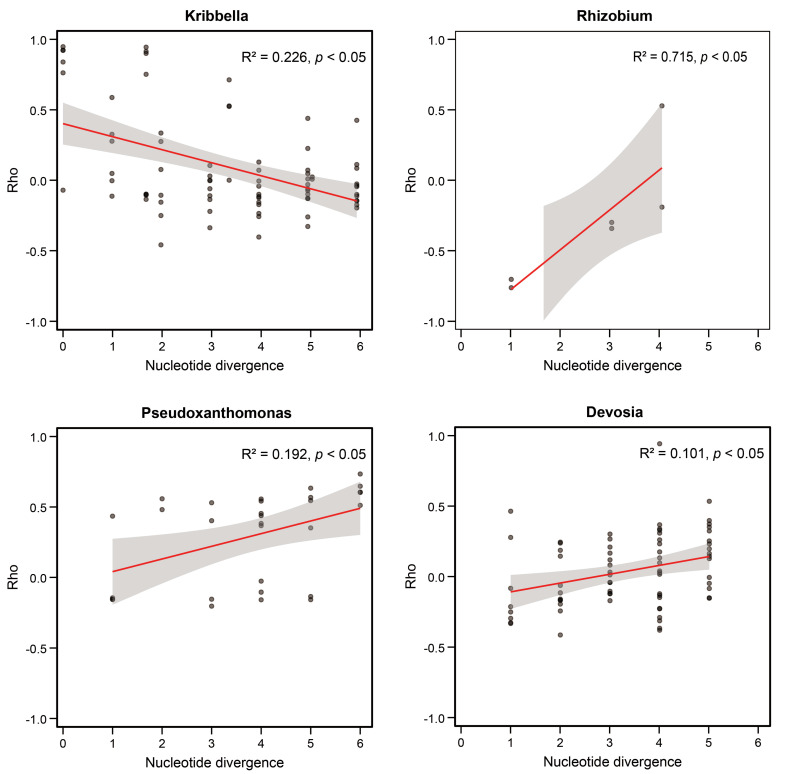
Relationship between proportionality-based covariation and nucleotide divergence within selected bacterial genera. Rho represents the proportionality value between OTU pairs and was used as a proxy for covariation or potential niche similarity among closely related taxa. The *x*-axis shows the number of nucleotide differences between OTU representative sequences. Each point represents one pairwise comparison between OTUs within the same genus. Only genera with three or more OTUs and fewer than six nucleotide differences were evaluated. The red line indicates the fitted linear relationship, and the gray band indicates the 95% confidence interval. R^2^ and *p* values are shown for significant regressions. OTU, operational taxonomic unit.

Among the 32 evaluated genera, *Devosia* (Alphaproteobacteria, Hyphomicrobiaceae), *Pseudoxanthomonas* (Gammaproteobacteria, Xanthomonadaceae), and Rhizobium (Alphaproteobacteria, Rhizobiaceae) exhibited a significant increase in the Rho ratio as nucleotide diversity increased (Figure 3). In contrast, Kribbella (Actinobacteria, Nocardioidaceae) exhibited a decrease in the Rho ratio. Devosia had the highest number of OTUs (41), with Rho scores ranging from 0.004 to 0.942. Rhizobium had the fewest OTUs (9), and its Rho distribution was similar to that of Pseudoxanthomonas (13).

### 3.3. Environmental Factors Driving Niche Differentiation Within Microbial Genera

**Figure 4:**
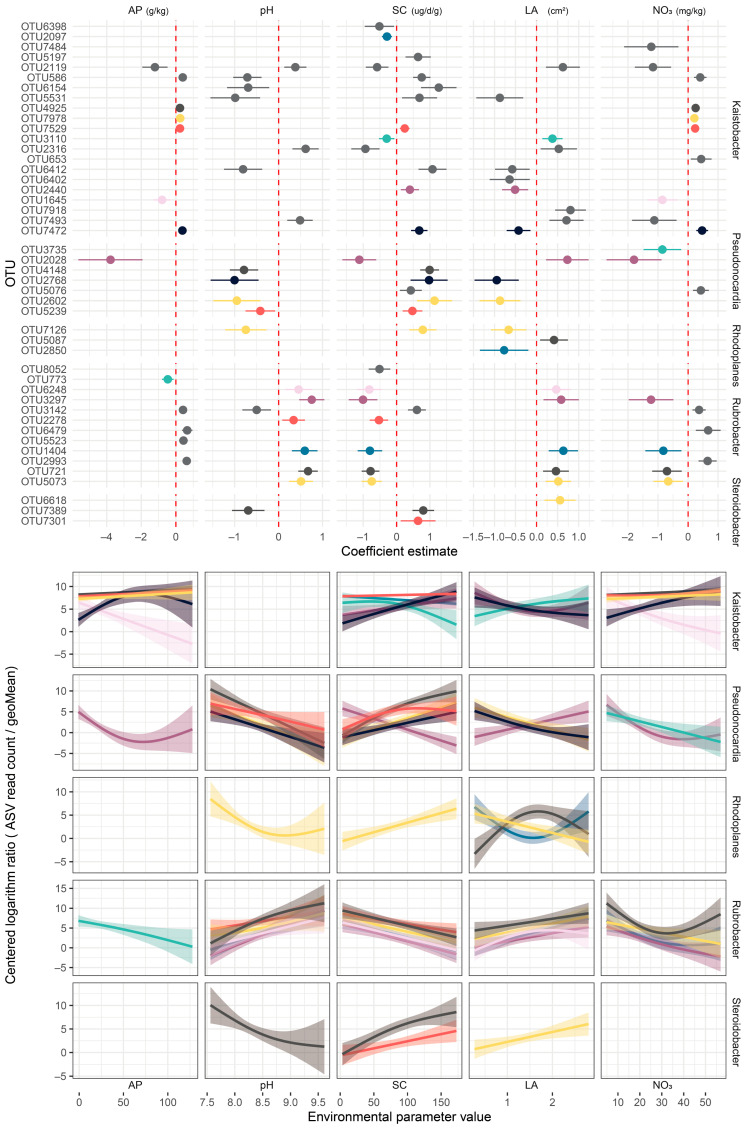
Responses of intragenus OTUs to environmental and rhizosphere activity variables. The upper panels show coefficient estimates from beta-binomial models for OTUs within the genera *Kaistobacter*, *Rubrobacter*, *Pseudonocardia*, *Rhodoplanes*, and *Steroidobacter*. Points indicate coefficient estimates, and horizontal bars indicate 95% confidence intervals. Positive and negative values indicate positive and negative associations with the corresponding environmental variable, respectively. The lower panels show generalized additive model (GAM) fits between centered log-ratio-transformed OTU abundance and selected environmental variables. Colors indicate different OTUs within each genus; only the eight most abundant OTUs are colored, and other OTUs are shown in gray. OTUs are ordered by hierarchical clustering based on nucleotide differences. UE, soil urease; AKP, soil alkaline phosphatase; EC, electrical conductivity; NH_4_^+^-N, ammonium nitrogen. Each composite rhizosphere soil sample was treated as one biological replicate (*n* = 48).

To investigate the effects of environmental factors on bacterial community diversity, we conducted phylogenetic and environmental response analyses across different operational taxonomic units (OTUs) within the dominant genera (the top five by abundance). Given the differences in altitudinal niches among taxonomic groups, we sought to assess how various environmental parameters influence these distributions. For each OTU-parameter pair, we investigated how the OTU responded (by increasing or decreasing in abundance) by generating models and estimating coefficients. Among the 704 response models, 258 showed significant responses (q ≤ 0.05; Figure 4, Supplementary Figure S2). Sixty-three percent of the models were linear, while the remainder were polynomial.

Analysis of nucleotide diversity within *Kaistobacter* reveals that nucleotide sequence differences between OTUs within the genus are extremely small (mostly ranging from 1 to 10 bases). This indicates that they constitute a group that is phylogenetically extremely closely related. However, the Corncob differential abundance model indicates that these closely related OTUs do not respond to the environment as a unified group. Within the genera *Kaistobacter*, *Pseudonocardia*, and *Rubrobacter*, the coefficient estimates for OTUs within the same genus show significant divergence on both sides of the zero axis. Using UE and AKP as examples, some OTUs within the genera *Kaistobacter*, *Pseudonocardia*, and *Rubrobacter* exhibited significant positive correlations, while others showed negative correlations. OTU6154 and OTU586 (nucleotide divergence of 5; Figure S3) were negatively correlated with pH and positively correlated with SC, AKP, and ammonia nitrogen. Curves from the Generalized Additive Model (GAM) further revealed the dynamic succession and nonlinear threshold characteristics of bacteria across environmental gradients. As environmental gradients such as UE, AKP, NH_4_, EC, AP, pH, and SC increased, multiple fitted curves within each genus exhibited an interdependent, crisscrossing pattern. For example, within a specific range of a given environmental parameter (such as UE or AKP), the abundance of one OTU declined sharply, while that of another OTU within the same genus rose rapidly. These relationships describe statistical covariation patterns between proportionality and sequence divergence and were therefore interpreted as indicators of potential differentiation in environmental responses rather than as direct evidence of ecological competition.

### 3.4. Relative Importance of Microbial Community Assembly Processes

**Figure 5:**
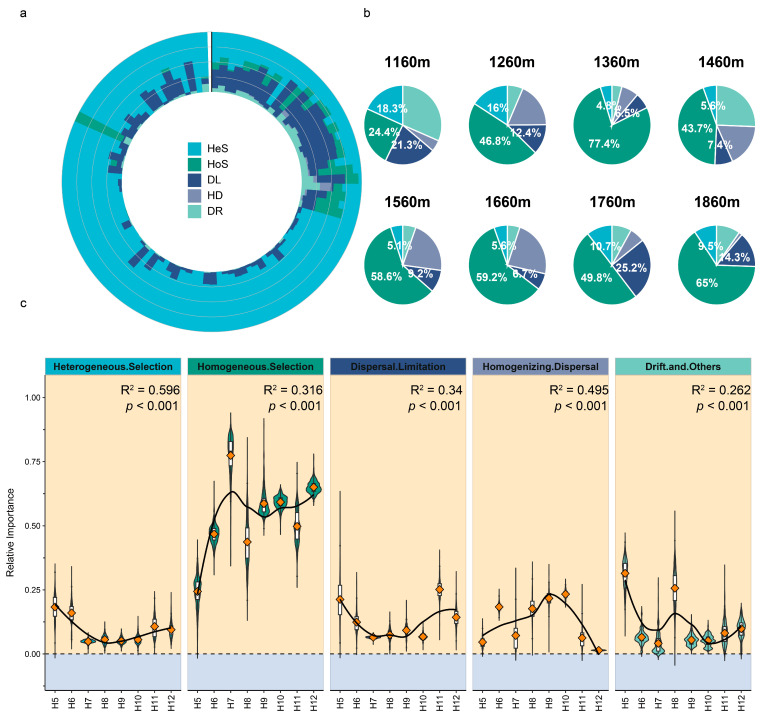
Relative importance of bacterial community assembly processes across the elevational gradient. (**a**) Relative importance of different assembly processes within each phylogenetic bin identified by iCAMP. (**b**) Mean relative importance of assembly processes at each elevation. (**c**) Elevation-related changes in the relative importance of assembly processes; violin plots and boxplots are based on 1000 bootstrap resamplings at each elevation level. Elevations ranged from 1160 to 1860 m. iCAMP, Infer Community Assembly Mechanisms by Phylogenetic-bin-based null model analysis; HeS, heterogeneous selection; HoS, homogeneous selection; DL, dispersal limitation; HD, homogenizing dispersal; DR, drift and other undominated processes. OTUs were grouped into 95 phylogenetic bins. Each composite rhizosphere soil sample was treated as one biological replicate (*n* = 48).

In the iCAMP analysis, the observed OTUs were classified into 95 phylogenetic groups (bins) based on phylogenetic signal thresholds, and the relative importance of different assembly processes was assessed separately for each group (Figure 5a). Overall, HeS dominated 78.9% of the bins, accounting for 32% of the relative abundance. In contrast, HoS accounted for only 5.26% of the bins, yet its relative abundance reached as high as 45.6%. Taxonomically diverse but low-abundance rare taxa are maintained by environmental spatial heterogeneity (HeS), while “low-diversity, ultra-high-abundance” core dominant communities are subject to extremely strong homogenizing selection (HoS) in the rhizosphere of host plants, thereby allowing HoS to dominate overall community turnover (abundance-weighted model). Neither HD nor DR overwhelmingly dominated other processes. When calculating the abundance of each bin, the influence of HeS (63%) was primarily attributed to the responses of Planctomycetia, Alphaproteobacteria, Actinobacteria, Spartobacteria, and Saprospirae. Overall, HoS dominated community succession and exhibited a strong relative influence across all elevation levels (averaging from 24.38% to 77.38%), corresponding to the functional and phylogenetic convergence of the rhizosphere microbial communities of *Carex enervis* at different elevations (Figure 5b,c).

**Figure 6:**
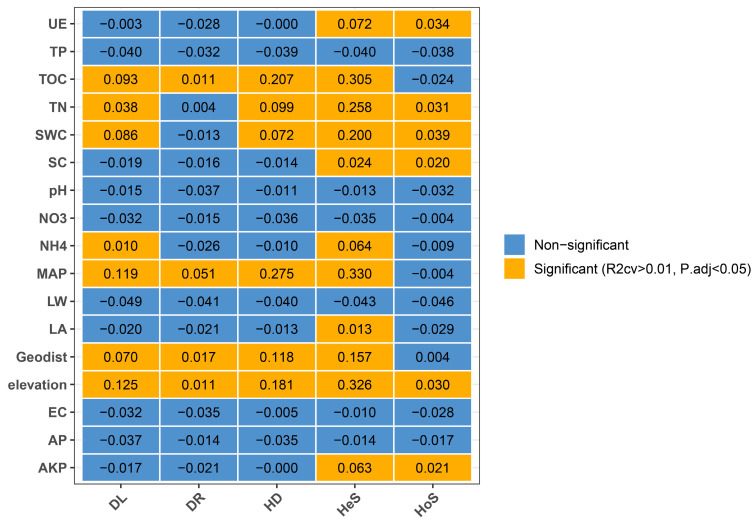
Associations between environmental variables and bacterial community assembly processes. The heatmap shows cross-validated coefficients of determination (R^2^_CV_) from Mantel-based association analysis between environmental variables and the relative importance of assembly processes. Yellow cells indicate significant associations (R^2^_CV_ > 0.01 and adjusted *p* < 0.05), whereas blue cells indicate non-significant associations. Assembly processes were estimated using iCAMP. UE, soil urease; TP, total phosphorus; TOC, total organic carbon; TN, total nitrogen; SWC, soil water content; SC, soil sucrase; pH, soil pH; NO_3_^−^-N, nitrate nitrogen; NH_4_^+^-N, ammonium nitrogen; MAP, mean annual precipitation; LW, longitude; LA, latitude; Geodist, geographic distance; EC, electrical conductivity; AP, available phosphorus; AKP, soil alkaline phosphatase; DL, dispersal limitation; DR, drift and other undominated processes; HD, homogenizing dispersal; HeS, heterogeneous selection; HoS, homogeneous selection. Each composite rhizosphere soil sample was treated as one biological replicate (*n* = 48).

To investigate the important roles that environmental factors and spatial variables play in influencing the aggregation of bacterial communities, we employed a cross-validated Mantel test to reveal the associations between each environmental variable and each assembly process. The estimated relative importance of different assembly processes was first converted to log-centered ratios for use in the Mantel test to address issues of data composition. Significant correlations (R^2^_CV_ > 0.01, *p* < 0.05) were visualized using a heatmap (Figure 6). Heterogeneous selection exhibited more and stronger correlations with the measured environmental variables, including elevation, MAP, SWC, and TOC, with an average R^2^_CV_ of 0.3 and a maximum R^2^_CV_ of 0.326. In contrast, DR was significantly correlated with only four environmental variables, including TOC and MAP, with R^2^_CV_ values all < 0.02.

Orthogonal partial least squares (OPLS) analysis was applied to identify the primary determinants of variation in each assembly process (Table S3). The OPLS models for the different processes were statistically significant (*p* ≤ 0.05, R^2^_Y_, the proportion of variance in the dependent variable Y explained by the model). They showed no obvious overfitting (*p* = 0.01, Q^2^_Y_, the model’s predictive accuracy). Elevation and mean annual precipitation (MAP) exhibited extremely high VIP values (all > 1.5) across nearly all community assembly processes. The climatic (particularly precipitation) and spatial isolation effects resulting from elevation gradients are the core drivers reshaping the structure and assembly mechanisms of the *Carex enervis* rhizosphere microbial community; meanwhile, soil physicochemical properties such as TOC and TN act as key local environmental filtering factors, synergistically regulating community assembly.

### 3.5. Associations Among Elevation, Rhizosphere Activity Proxies, Microbial Response Groups, and Nitrogen Availability

To evaluate hypothesized associations among elevation, enzyme-based proxies of rhizosphere activity, microbial response groups, and ammonium nitrogen availability, this study constructed a partial least squares path model (PLS-PM). The evaluation results of the measurement model confirmed that the latent variable “Plant Activity” is accurately characterized by sucrase (SC), urease (UE), and alkaline phosphatase (AKP), with external loadings for each observed indicator exceeding 0.78 (ranging from 0.784 to 0.884). The overall goodness-of-fit (GoF) of this mediating model reached 0.544, effectively explaining the driving pathways of the rhizosphere microbial network in *Carex enervis*.

**Figure 7:**
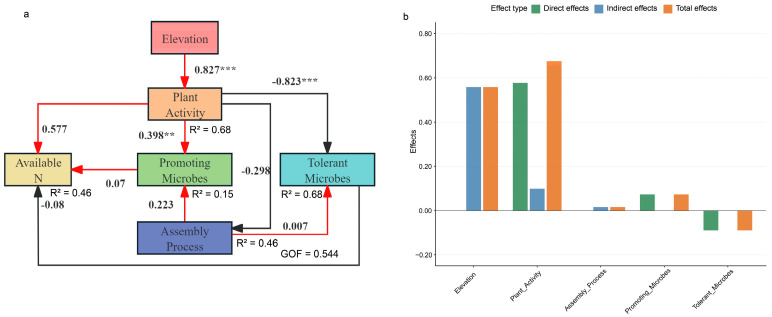
PLS-PM-based association framework linking elevation, rhizosphere activity proxies, microbial response groups, assembly processes, and ammonium nitrogen availability. (**a**) The model evaluates hypothesized statistical associations among elevation, enzyme-based proxies of rhizosphere activity, heterogeneous selection, microbial response groups, and ammonium nitrogen availability. Rhizosphere activity was represented by soil sucrase (SC), soil urease (UE), and soil alkaline phosphatase (AKP). Positively associated taxa refer to OTUs positively associated with rhizosphere activity proxies, whereas negatively associated or less responsive taxa refer to OTUs showing negative or weak associations with these proxies. Arrows indicate standardized path coefficients within the PLS-PM framework. Red and black arrows indicate positive and negative associations, respectively. R^2^ values indicate the proportion of variance explained for endogenous variables. Path coefficient significance was assessed using 1000 bootstrap resamplings. PLS-PM, partial least squares path modeling; HeS, heterogeneous selection; NH_4_^+^-N, ammonium nitrogen. These paths represent statistical associations and should not be interpreted as experimentally verified causal effects. The model included samples with complete data on environmental conditions, enzyme activity, microbial abundance, and NH_4_^+^-N (*n* = 48). ** *p* < 0.01, *** *p* < 0.001. (**b**) Standardized direct, indirect, and total effects of each latent variable on available nitrogen estimated by the PLS-PM. The model describes statistical associations among variables and should not be interpreted as evidence of direct biological causality.

The model indicates that elevation serves as the macro-level driver of this microecological network, exerting a significant positive influence on plant rhizosphere activity (path coefficient = 0.827) and accounting for 68.45% of its variance (Figure 7a). Higher values of enzyme-based rhizosphere activity proxies were associated with a lower relative importance of heterogeneous selection (HeS; path coefficient = −0.298). These rhizosphere activity proxies were also associated with differences in the relative abundance of closely related microbial taxa. On the one hand, it was significantly associated with the enrichment of “positively associated taxa” (*Kaistobacter*-positive response groups) (path coefficient = 0.398); on the other hand, it was negatively associated with the competitively disadvantaged “environmentally tolerant taxa” (*Kaistobacter*-negative response groups and *Rubrobacter*) (path coefficient = −0.823), explaining 68.08% of the abundance variation in this tolerance group. Rhizosphere activity proxies showed a direct positive path coefficient with ammonium nitrogen (NH_4_^+^-N) availability in the PLS-PM framework (path coefficient = 0.577). The entire PLS-PM model collectively explained 46.41% of the variation in the rhizosphere available nitrogen pool (Figure 7b).

PICRUSt2-based functional prediction indicated that the predicted nitrogen metabolism potential varied across the elevational gradient. The predicted abundance of nitrogen metabolism was relatively higher at 1150 m and declined at higher elevations (Figure S4a). Module-level profiles showed that ammonia assimilation accounted for the largest proportion of predicted nitrogen metabolism functions. In contrast, nitrogen fixation-related KOs represented only a subset of the predicted functional profile (Figure S4b). Heatmap analysis further showed elevation-associated variation in several nitrogen metabolism-related KOs (Figure S4c).

## 4. Discussion

### 4.1. Host-Based Homogenization Filtering and Environmental Heterogeneity Jointly Shape Rhizosphere Community Assembly

Unraveling the mechanisms of community assembly is central to understanding the formation of rhizosphere microbial communities. iCAMP analysis revealed a “dual-track” assembly pattern: although heterogeneous selection (HeS) was overwhelmingly dominant in the number of phylogenetic groups (bins) (78.9%), homogeneous selection (HoS) dominated in relative abundance (45.6%). This phenomenon has profound ecological implications. On the one hand, rare or associated microbial communities characterized by “high diversity and low abundance” are primarily governed by environmental spatial heterogeneity (HeS) [33]. Mantel tests further confirm that gradient changes in macro-environmental factors—such as elevation, mean annual precipitation (MAP), soil water content (SWC), and total organic carbon (TOC)—provide diverse spatiotemporal niches for these widespread or rare species. On the other hand, the rhizosphere of the host plant, *Carex enervis*, acts as a strong “environmental filter.” Despite drastic changes in the external altitudinal environment, the core dominant microbial community, driven by homogeneous selection (HoS), maintains a strong relative influence across all altitudinal levels. This finding is consistent with previous community assembly studies showing that different microbial lineages within the same community may be governed by different ecological processes, supporting the use of bin-based approaches such as iCAMP to resolve lineage-specific assembly patterns. The rhizosphere of *Carex enervis* may provide relatively stable microhabitat conditions across elevations, which were associated with the abundance-weighted contribution of homogeneous selection among core dominant taxa [34,35,36]. The physical structure of the root surface (such as water-holding capacity) inherently provides a homogeneous habitat. At the same time, homogenization of filtration may determine which organisms become the true “dominators” of the rhizosphere. Together, these two factors maintain the stability of rhizosphere communities in alpine meadows.

### 4.2. Fine-Scale Differentiation in Covariation Patterns Among Closely Related Taxa

This study identified highly resolved variation in niche preferences within core genera, revealing sophisticated strategies by which microorganisms avoid competition in alpine habitats. Traditional hypotheses of phylogenetic conservatism suggest that species with closer phylogenetic relationships share more similar niches [37,38,39,40]. Our results observed this classic pattern within the genus *Kribbella* (where Rho values decrease as nucleotide divergence increases). However, in dominant genera such as *Devosia*, *Rhizobium*, and *Pseudoxanthomonas*, we discovered a seemingly counterintuitive pattern of significant positive correlation. As nucleotide distance increases, the covariance (Rho) between closely related taxa actually rises significantly. Similar amplicon-based studies have reported that closely related bacterial ASVs or OTUs within the same genus can show divergent temporal or environmental response patterns, suggesting that genus-level taxa may contain ecologically differentiated lineages.

This pattern provides evidence that closely related taxa within the same genus may show divergent covariation patterns along environmental gradients. Closely related taxa with similar ecological preferences may respond differently to local rhizosphere conditions under limited resource availability, thereby contributing to differences in their relative abundances across microhabitats [41,42]. The GAM-based abundance curves further suggest contrasting environmental response patterns among intragenus taxa: across the same environmental gradients (e.g., UE, AKP), the abundance curves of closely related OTUs within the same genus exhibit a typical cross-substitution pattern, in which one gains at the expense of the other. The relationship between Rho and nucleotide divergence indicates divergent covariation patterns among intragenus taxa but does not directly demonstrate competitive exclusion [43,44,45,46,47]. This pattern suggests that core rhizosphere microbial taxa in alpine meadows may exhibit fine-scale niche-related differentiation across environmental gradients, potentially contributing to community turnover amid complex environmental fluctuations.

### 4.3. Associations Between Rhizosphere Activity Proxies, Microbial Response Groups, and Ammonium Nitrogen Availability

Dynamic changes in rhizosphere microorganisms may contribute to the survival and growth of host plants. To gain a deeper understanding of the potential association pathways involved in this process, a PLS-PM model was constructed to evaluate this complex interaction network. The model indicated that elevation, as a macro-scale environmental factor, was not only associated with changes in the physical environment but was also positively linked to plant rhizosphere activity, as represented by SC, UE, and AKP (R^2^ = 0.68). It is worth noting that this study used the activities of SC, UE, and AKP as proxies for rhizosphere activity. Although soil extracellular enzymes are a mixture of plant root exudates and soil microbial metabolites, which makes them difficult to separate physically, this does not preclude their use as useful indicators of the intensity of rhizosphere interactions. Previous studies have shown that the “rhizosphere stimulation effect” triggered by plant carbon input via the root system is the primary driving force behind the disruption of soil baseline equilibrium [48,49,50]. Therefore, the increase in enzyme activity in the model may reflect a coordinated rhizosphere process in which plant-related inputs and microbial activity jointly reshape the microhabitat, potentially helping to alleviate nutrient limitations in alpine habitats [51,52,53].

With this enhanced rhizosphere activity, the rhizosphere of *Carex enervis* appeared to function as a microbiologically active microenvironment. Potentially through plant-derived carbon inputs and enzyme-associated processes, it was associated with shifts in the soil’s inherent assembly patterns, including a reduced relative importance of heterogeneous selection (HeS) (path coefficient = −0.298). Accompanying this shift in assembly patterns, the model suggested differential associations among microbial response groups: rhizosphere activity was positively associated with a “positively associated taxa” group represented by certain *Kaistobacter* species, while showing a negative association with the “tolerant/suppressed microbial groups” (path coefficient = −0.823). Previous studies have indicated that in alpine mountain meadows, where readily available nutrients such as nitrogen and phosphorus are severely limited, plants generally rely on the synergistic action of rhizosphere microorganisms to maintain nutrient supply [54,55,56,57]. The model confirms that rhizosphere activity and the resulting changes in microbial communities are the most critical drivers of the increase in readily available nitrogen (NH_4_) content (R^2^ = 0.46). These results suggest that in alpine mountain meadows with limited nutrient availability, the rhizosphere of *Carex enervis* may provide a relatively stable microenvironment within a heterogeneous macrohabitat, where specific microbial response groups and niche-differentiated, closely related taxa are associated with enhanced availability of ammonium nitrogen.

### 4.4. Limitations

A key limitation of this study is that it was based on an observational elevational gradient. Although the results revealed associations among elevation, proxies of rhizosphere activity, bacterial community assembly, and ammonium nitrogen availability, they cannot demonstrate plant-mediated microbial attraction, recruitment, or exclusion. In addition, the study was conducted over a single growing season; therefore, seasonal or interannual variation in rhizosphere bacterial assembly could not be evaluated. Because the microbial analysis was based on 16S rRNA gene amplicon sequencing, the taxonomic resolution was limited, and the inferred functional patterns should be interpreted cautiously. Future studies combining plant removal, root exudate analysis or addition, microbial inoculation, metagenomics, metatranscriptomics, and culture-based validation are needed to test these causal mechanisms directly. Although PICRUSt2 predicted the presence of nitrogen fixation-related KOs, including nifH, nifD, and nifK, these predictions should be interpreted only as putative functional potential. Because nifH expression, nifH gene abundance, nitrogenase activity, and direct nitrogen fixation rates were not measured, this study cannot demonstrate active nitrogen fixation. Direct validation using nifH qPCR/RT-qPCR, nitrogenase activity assays, metagenomics/metatranscriptomics, or ^15^N_2_-based measurements would be required to confirm nitrogen fixation activity.

## 5. Conclusions

This study focuses on community assembly and plant–microbe co-adaptation mechanisms, revealing the characteristics of rhizosphere microbial community assembly in *Carex enervis* in alpine mountain meadows: Macroenvironmental heterogeneity, such as elevation and precipitation, was associated with rare microbial communities mainly through heterogeneous selection (HeS), whereas abundance-weighted patterns suggested homogeneous selection (HoS) of core dominant microbial communities in the rhizosphere. The results suggest that core dominant intragenus taxa in the rhizosphere exhibited fine-scale differentiation in environmental response patterns. These patterns may contribute to the coexistence and turnover of closely related taxa under heterogeneous alpine meadow conditions. Still, they should be interpreted as association-based evidence rather than direct proof of competitive exclusion or experimentally verified functional differentiation. Along the elevational gradient, rhizosphere conditions of *Carex enervis* were associated with shifts in microbial response groups and ammonium nitrogen availability, suggesting potential links between bacterial community assembly and nutrient-related soil processes. This study enriches our understanding of the mechanisms underlying microecological niche differentiation in closely related species. It provides critical theoretical support for understanding how arid and semi-arid mountain ecosystems mitigate macro-stressors and maintain nutrient cycling through plant–microbe interactions at the micro-scale under climate change.

## Data Availability

The data presented in this study are available on request from the corresponding author. The data are not publicly available due to privacy.

## Supplementary Materials

The following supporting information can be downloaded at: https://www.mdpi.com/article/10.3390/microorganisms14071468/s1, Table S1: Dominant plant species and their relative coverage at different sampling sites across the elevational gradient. Table S2: Simper analysis results showed the contribution of dominant bacteria to 16S rRNA sequences. Table S3: Performance indicators of each assembly process and environmental variables in the orthogonal partial least squares (OPLS) model. Figure S1: Bacterial community alpha diversity on the gradient (richness estimate calculated based on the breakaway package) compared with NMDS analysis. Figure S2: Significant model analysis of bacterial communities between genera and environmental parameters. Figure S3: Nucleotide heatmap of the genera Kaistobacter, Pseudonocardia, Rubrobacter, Rubrobacter, and Steroidobacter. Figure S4: PICRUSt2-predicted nitrogen metabolism functional potential across elevations.

## Abbreviations

The following abbreviations are used in this manuscript:

MAP	Mean annual precipitation
SWC	Soil moisture content
EC	Electrical conductivity
SOC	Soil organic matter
NO_3_^−^-N	Nitrate nitrogen
NH_4_^+^-N	Ammonium nitrogen
TN	Soil total nitrogen
TP	Soil total phosphorus
AP	Soil available phosphorus
AKP	Soil alkaline phosphatase
UE	Soil urease
SC	Soil sucrase
LA	Leaf area
LW	Leaf wide
HeS	Heterogeneous selection
HoS	Homogeneous selection
DL	Dispersal limitation
HD	Homogenizing dispersion
DR	Drift
