# Supplementary material for: Elevational Variation in Rhizosphere Bacterial Assembly and Fine-Scale Taxon Differentiation of Carex enervis in Arid and Semi-Arid Alpine Meadows
Source: Microorganisms. 2026 Jul 3;14(7):1468. doi: 10.3390/microorganisms14071468 (PMC13414172; doi:10.3390/microorganisms14071468)

Table S1: Dominant plant species and their relative coverage at different sampling sites across the e levational gradient.

Elevation （m）	Dominant species			
	Sloping land	Relative coverage	Flat ground	Relative coverage
860m	*Salsola rosacea* L.	74%	*Petrosimonia squarrosa (Schrenk) Bunge*	68%
960m	*Salsola rosacea* L.	70%	*Halimocnemis villosa*	77%
1060m	*Halimocnemis villosa*	84%	*Petrosimonia squarrosa (Schrenk) Bunge*	68%
1160m	*Salsola rosacea* L.	78%	*Seriphidium heptapotamicum (Poljakov)* Y. Ling & Y. R. Ling	72%
1260m	*Carex enervis* C. A. Mey	66%	*Dysphania botrys (Linnaeus)* Mosyakin & Clemants	54%
1360m	*Carex enervis* C. A. Mey	55%	*Carex enervis* C. A. Mey	65%
1460m	*Carex enervis* C. A. Mey	60%	*Carex enervis* C. A. Mey	68%
1560m	*Carex enervis* C. A. Mey	68%	*Polygonum aviculare* L.	55%
1660m	*Carex enervis* C. A. Mey	60%	*Carex enervis* C. A. Mey	58%
1760m	*Carex enervis* C. A. Mey	59%	*Carex enervis* C. A. Mey	64%
1860m	*Carex enervis* C. A. Mey	55%	*Carex enervis* C. A. Mey	54%

Table S2: Simper analysis results showed the contribution of dominant bacteria to 16S rRNA seque nces.

OTU	Contribution	Phylum	Genus	Sequence
OTU3980	34.91%	Verrucomicrobia	DA101	AAACCCTGAAGGAGCGACGCCGCGTGGAGGATGAAGGCTTTCGGGTTGTAAACTCCTGTCATTTGAGAACAAGGCGCCGATATTAACTGTATCGGCGTTGATAGTATCAGAAGAGGAAGGGACGGCTAACTCTGTGCCAGCAGCCGCGGTAATACAGAGGTCCCAAGCGTTGTTCGGATTCATTGGGCGTAAAGGGTGCGTAGGTGGCGCCGTAAGTCGGGTGTGAAATTTCGGAGCTTAACTCCGAAACTGCATTCGATACTGCGGTGCTTGAGGACTGGAGAGGAGACTGGAATTCATGGTGTAGCAGTGAAATGCGTAGAGATCATGAGGAAGACCAGTGGCGAAGGCGGGTCTCTGGACAGTTCCTGACACTG
OTU193	32.35%	Firmicutes	Bacillus	AAAGTCTGACGGAGCAACGCCGCGTGAACGAAGAAGGCCTTCGGGTCGTAAAGTTCTGTTGTTAGGGAAGAACAAGTACCAGAGTAACTGCTGGTACCTTGACGGTACCTAACCAGAAAGCCACGGCTAACTACGTGCCAGCAGCCGCGGTAATACGTAGGTGGCAAGCGTTGTCCGGAATTATTGGGCGTAAAGCGCGC

				GCAGGTGGTTCCTTAAGTCTGATGTGAAAGCCCACGGCTC AACCGTGGAGGGTCATTGGAAACTGGGAACTTGAGTGCA GAAGAGGAAAGTGGAATTCCAAGTGTAGCGGTGAAATGC GTAGAGATTTGGAGGAACACCAGTGGCGAAGGCGACTTTC TGGTCTGTAACTGACACTG
OTU318	29.74%	Verrucomicrobia	DA101	AAACCCTGAAGGAGCGACGCCGCGTGGAGGATGAAGGCT TTCGGGTTGTAAACTCCTGTCATTTGAGAACAAGGTGCACC GGTTAACTGCCGGTGCATTGATAGTATCAGAAGAGGAAGG GACGGCTAACTCTGTGCCAGCAGCCGCGGTAATACAGAGG TCCCAAGCGTTGTTCGGATTCATTGGGCGTAAAGGGTGCGT AGGTGGCGCCGTAAGTCGGGTGTGAAATTTCGGAGCTTAA CTCCGAAACTGCATTCGATACTGCGGTGCTTGAGGACTGG AGAGGAGACTGGAATTCATGGTGTAGCAGTGAAATGCGTA GAGATCATGAGGAAGACCAGTGGCGAAGGCGGGTCTCTG GACAGTTCCTGACACTG
OTU7943	22.68%	Proteobacteria	Pseudomonas	AAAGCCTGATCCAGCCATGCCGCGTGTGTGAAGAAGGTCT TCGGATTGTAAAGCACTTTAAGTTGGGAGGAAGGGCAGTT ACCTAATACGTAATTGTTTTGACGTTACCGACAGAATAAGC ACCGGCTAACTCTGTGCCAGCAGCCGCGGTAATACAGAGG GTGCAAGCGTTAATCGGAATTACTGGGCGTAAAGCGCGCG TAGGTGGTTCGTTAAGTTGGATGTGAAATCCCCGGGCTCAA CCTGGGAACTGCATTCAAAACTGTCGAGCTAGAGTATGGT AGAGGGTGGTGGAATTTCCTGTGTAGCGGTGAAATGCGTA GATATAGGAAGGAACACCAGTGGCGAAGGCGACCACCTG GACTGATACTGACACTG
OTU4925	21.81%	Proteobacteria	Kaistobacter	AAAGCCTGATCCAGCAATGCCGCGTGAGTGATGAAGGCCT TAGGGTTGTAAAGCTCTTTTACCCGGGATGATAATGACAGT ACCGGGAGAATAAGCCCCGGCTAACTCCGTGCCAGCAGCC GCGGTAATACGGAGGGGGCTAGCGTTGTTCGGAATTACTG GGCGTAAAGCGTACGTAGGCGGCTTTGTAAGTTAGAGGTG AAAGCCCGGGGCTCAACTCCGGAATTGCCTTTAAGACTGC ATCGCTTGAATCATGGAGAGGTGAGTGGAATTCCGAGTGT AGAGGTGAAATTCGTAGATATTCGGAAGAACACCAGTGGC GAAGGCGACTCACTGGACATGTATTGACGCTG
OTU721	18.15%	Actinobacteria	Rubrobacter	AAAGCCTGACGCAGCAACACCGTGTGAGCGACGAAGGCC TTCGGGTCGTAAAGCTCTGTTGTTGGGGACGAAGGGTTAGG GGTTAATAGCCCCGAGCCTGACGGTACCCTTCGAGGAAGC CCCGGCTAACTACGTGCCAGCAGCCGCGGTAATACGTAGG GGGCGAGCGTTGTCCGGAATTATTGGGCGTAAAGAGCGTG TAGGCGGTTCGGTAAGTCTGCCGTGAAAACCCAGGGCTCA ACCCTGGGCGTGCGGTGGATACTGCCGGGCTAGAGGGTGG TAGAGGCGAGTGGAATTCCCGGTGTAGCGGTGAAATGCGC AGATATCGGGAGGAACACCAGTAGCGAAGGCGGCTCGCT GGGCCACACCTGACGCTG
OTU6721	15.78%	Firmicutes	Bacillus	AAAGTCTGACGGAGCAACGCCGCGTGAGCGATGAAGGCC TTCGGGTCGTAAAGCTCTGTTGTTAGGGAAGAACAAGTATC GGAGTAACTGCCGGTACCTTGACGGTACCTAACCAGAAAG CCACGGCTAACTACGTGCCAGCAGCCGCGGTAATACGTAG GTGGCAAGCGTTGTCCGGAATTATTGGGCGTAAAGCGCGC GCAGGCGGTCCTTTAAGTCTGATGTGAAAGCCCACGGCTC AACCGTGGAGGGTCATTGGAAACTGGGGGACTTGAGTACA

				GAAGAGGAAAGCGGAATTCCACGTGTAGCGGTGAAATGCGTAGAGATGTGGAGGAACACCAGTGGCGAAGGCGGCTTTCTGGTCTGTAACTGACGCTG
OTU2278	13.45%	Actinobacteria	Rubrobacter	AAAGCCTGACCCAGCAACACCGTGTGGGCGATGAAGGCCTTCGGGTCGTAAAGCCCTGTTGATAGGGACGAAGGGCGAAGGGTTAATAGCCCCTAGCCTGACGGTACCTTTCGAGGAAGCCCCGGCTAACTACGTGCCAGCAGCCGCGGTAATACGTAGGGGGCGAGCGTTGTCCGGAATTATTGGGCGTAAAGAGCGTGTAGGCGGTTCGGTAAGTCTGCCGTGAAAACCTGAGGCTCAACCTCGGGCGTGCGGTGGATACTGCCGGGCTAGAGGACGGTAGAGGCGAGTGGAATTCCCGGTGTAGCGGTGAAATGCGCAGATATCGGGAGGAACACCAGTAGCGAAGGCGGCTCGCTGGGCCGTTCCTGACGCTG
OTU5439	12.37%	Proteobacteria	Bradyrhizobium	CAAGCCTGATCCAGCCATGCCGCGTGAGTGATGAAGGCCCTAGGGTTGTAAAGCTCTTTTGTGCGGGAAGATAATGACGGTACCGCAAGAATAAGCCCCGGCTAACTTCGTGCCAGCAGCCGCGGTAATACGAAGGGGGCTAGCGTTGCTCGGAATCACTGGGCGTAAAGGGTGCGTAGGCGGGTCTTTAAGTCAGGGGTGAAATCCTGGAGCTCAACTCCAGAACTGCCTTTGATACTGAAGATCTTGAGTTCGGGAGAGGTGAGTGGAACTGCGAGTGTAGAGGTGAAATTCGTAGATATTCGCAAGAACACCAGTGGCGAAGGCGGCTCACTGGCCCGATACTGACGCTG
OTU4500	11.49%	Firmicutes	Bacillus	AAAGTCTGACGGAGCAACGCCGCGTGAGTGATGAAGGCTTTCGGGTCGTAAAACTCTGTTGTTAGGGAAGAACAAGTGCTAGTTGAATAAGCTGGCACCTTGACGGTACCTAACCAGAAAGCCACGGCTAACTACGTGCCAGCAGCCGCGGTAATACGTAGGTGGCAAGCGTTATCCGGAATTATTGGGCGTAAAGCGCGCGCAGGTGGTTTCTTAAGTCTGATGTGAAAGCCCACGGCTCAACCGTGGAGGGTCATTGGAAACTGGGAGACTTGAGTGCAGAAGAGGAAAGTGGAATTCCATGTGTAGCGGTGAAATGCGTAGAGATATGGAGGAACACCAGTGGCGAAGGCGACTTTCTGGTCTGTAACTGACACTG

Table S3: Performance indicators of each assembly process and environmental variables in the orthogonal partial least squares (OPLS) model. R^2_Y and R^2_X represent the percentage of Y (i.e., relative importance of assembly process) and X (i.e. environmental variables) dispersion (i.e., sum of squares) explained by the model, respectively; Q^2_Y reflects the overall predictive performance of the model; P_{Q2Y} and P_{R2Y} reflect the significance of Q^2Y and R^2_Y compared to the results from random permutations; VIP, variable importance in projection, of which the values >1 point to the most relevant variables.

	Heterogeneous Selection	Homogeneous Selection	Dispersal Limitation	Homogenizing Dispersal	Drift and Others
Q^2_Y	0.455	0.148	0.169	0.322	0.0904
P_{Q2Y}	0.01	0.01	0.01	0.01	0.01
R^2_Y	0.462	0.162	0.184	0.335	0.105
P_{R2Y}	0.01	0.01	0.01	0.01	0.01
R^2X	0.447	0.439	0.46	0.447	0.436
Variable	VIP	VIP	VIP	VIP	VIP
Geodist	**1.21**	0.22	**1.19**	**1.44**	**1.42**
MAP	**1.71**	0.54	**1.74**	**1.90**	**1.55**
TN	**1.37**	**1.48**	**1.25**	**1.22**	**1.17**
AP	0.29	**1.21**	0.17	0.13	**1.20**
TOC	**1.44**	0.91	**1.36**	**1.46**	**1.32**
pH	0.45	0.35	0.61	0.46	0.28
SC	0.65	**1.47**	0.55	0.50	0.98
UE	**1.04**	**1.52**	0.97	0.82	0.84
AKP	0.86	**1.52**	0.76	0.66	0.92
SWC	**1.39**	**1.16**	**1.53**	**1.34**	0.88
LA	0.51	0.74	0.31	0.36	0.43
EC	0.55	0.93	0.37	0.78	0.45
NH_4	0.84	0.66	**1.01**	0.65	0.32
NO_3	0.16	**1.07**	0.20	0.06	**1.21**
TP	0.09	0.44	0.49	0.12	0.91
LW	0.02	0.12	0.01	0.06	0.30
elevation	**1.69**	0.86	**1.75**	**1.77**	**1.34**

Figure S1: Bacterial community alpha diversity on the gradient (richness estimate calculated based on the breakaway package) compared with NMDS analysis.

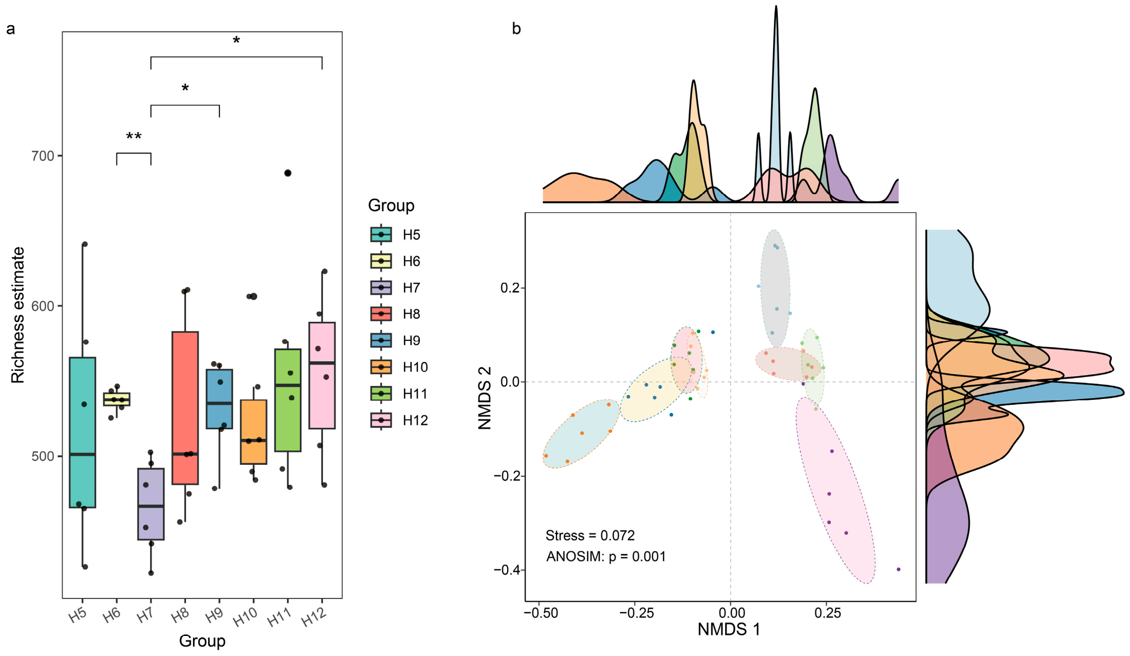

Figure S2: Significant model analysis of bacterial communities between genera and environmental p arameters. Coefficient estimates indicate positive or negative responses to the parameters, with 95% confidence intervals annotated. Colors correspond to different OTUs within the genus (only the top 8 high-abundance OTUs are labeled, the rest are shown in gray). OTUs are ordered based on hierar chical clustering analysis of nucleotide differences. The figure below shows the generalized additive model fit results, depicting the relationship between log ratio abundance centered on OTUs and the distribution of significant OTU parameter values indicated in the figure above. Subplots and OTU c olor distributions are consistent with those in the figure above.

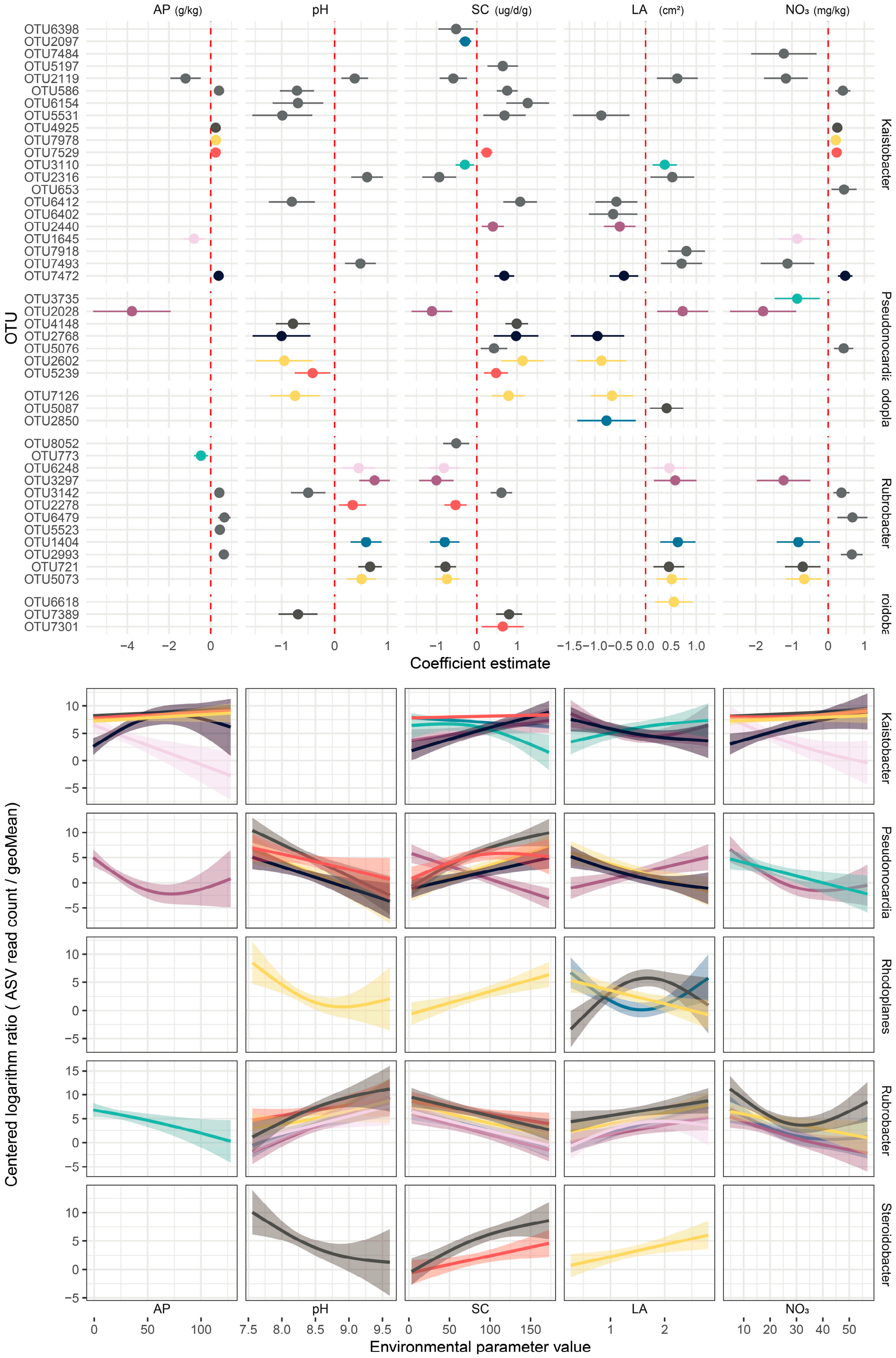
AP (g/kg)
pH
SC (ug/d/g)
LA (cm²)
NO₃ (mg/kg)
OTU
Kaistobacter
Pseudonocardia
Rhodoplanes
Rubrobacter
Steroidobacter
Coefficient estimate
Centered logarithm ratio (ASV read count / geoMean)
AP
pH
SC
LA
NO₃
Environmental parameter value

Figure S3: Nucleotide heatmap of the genera Kaistobacter, Pseudonocardia, Rubrobacter, Rubrobacter, and Steroidobacter. The intensity of the color corresponds to the abundance differences of different OTUs within each genus (only the top 8 high-abundance OTUs are colored, while the remaining OTUs are shown in gra y). The differentiation of 6 nucleotide positions corresponds to a median of 98.5% sequence identity in the 16S rRNA gene.

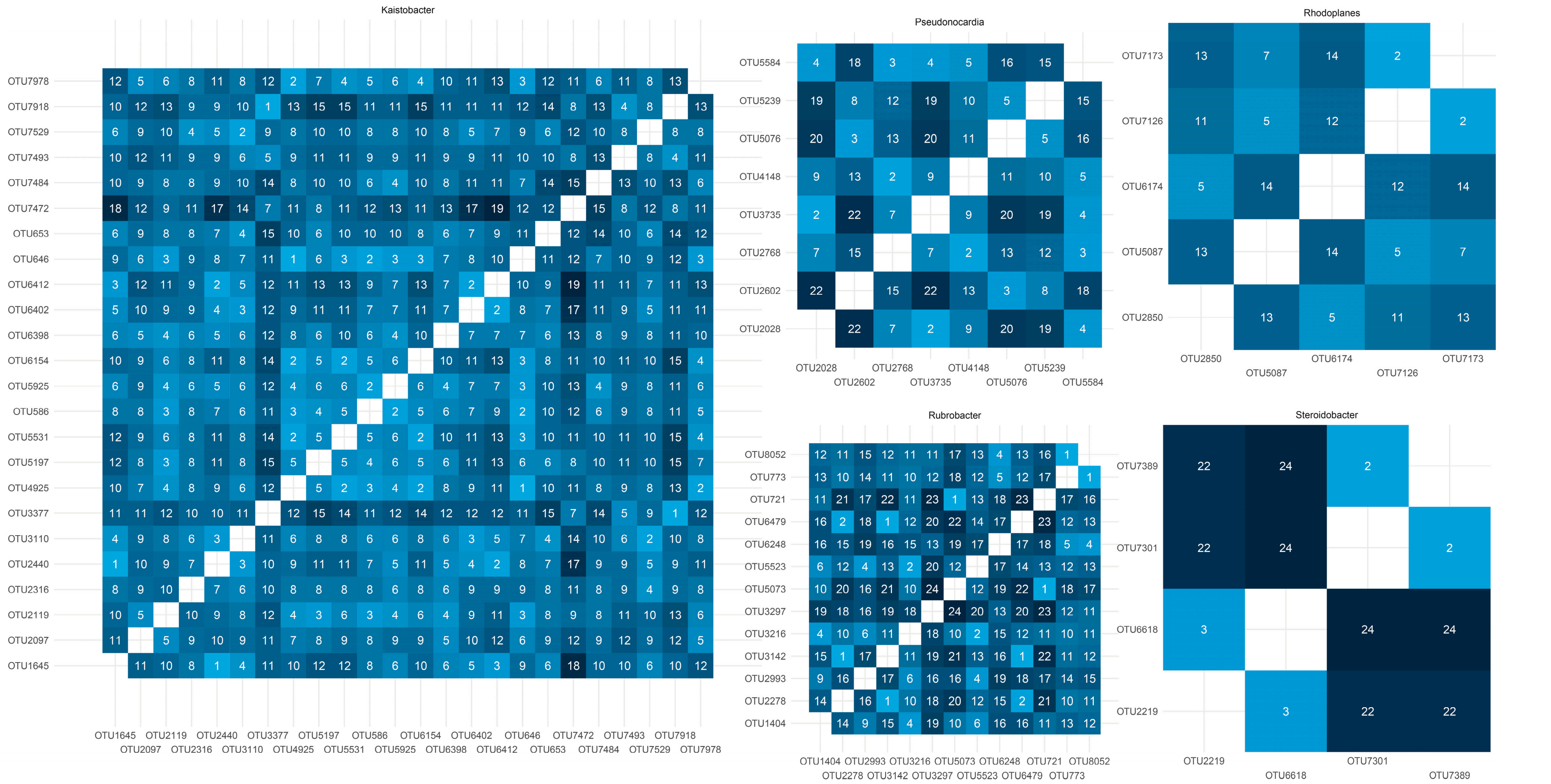

Figure S4: PICRUSt2-predicted nitrogen metabolism functional potential across elevations. (a) Predicted nitrogen metabolism abundance normalized per 10,000 predicted gene copies. (b) Relative abundance of predicted nitrogen metabolism modules across elevations. (c) Heatmap of Z-score-transformed predicted KO abundances associated with nitrogen metabolism. nifH, nifD, and nifK were included as predicted nitrogen fixation-related KOs. These results represent predicted functional potential inferred from 16S rRNA gene data and do not indicate gene expression or measured nitrogen fixation activity.

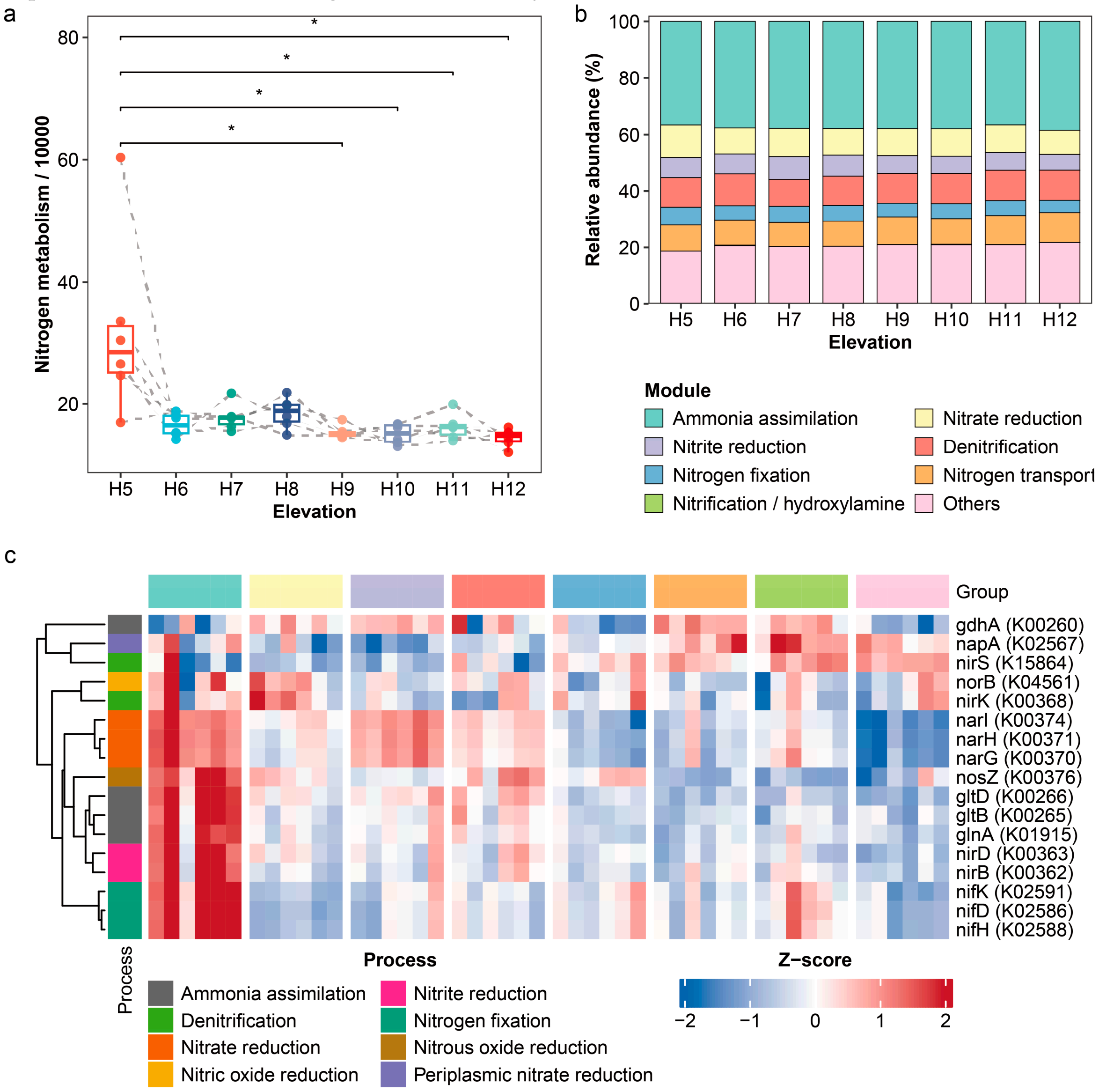

Supplement: Supplementary file 1 [file microorganisms-14-01468-s001.zip › microorganisms-4368641-supplementary.pdf]
